# Testosterone Therapy in Men in Their 40s: A Narrative Review of Indications, Outcomes, and Mid-Term Safety

**DOI:** 10.7759/cureus.92778

**Published:** 2025-09-20

**Authors:** Luis M Canal de Velasco, José Emiliano González Flores

**Affiliations:** 1 Medicine, Panamerican University, Mexico City, MEX; 2 Medicine, Tecnológico de Monterrey, Campus Ciudad de México, Mexico City, MEX

**Keywords:** bone mineral density, late-onset hypogonadism, metabolic syndrome, middle-aged men, sexual function improvement, testosterone replacement therapy

## Abstract

Late-onset testosterone deficiency in early middle-aged men (40-49 years) is increasingly recognized and is often associated with obesity, metabolic syndrome, and sleep apnea. Prevalence estimates suggest that 6%-12% of men in this age group have biochemically low testosterone levels, though variation in diagnostic thresholds, study populations, and assay methods contributes to differing estimates. This narrative review synthesizes current literature on the indications, clinical benefits, and mid-term safety of testosterone replacement therapy (TRT) in men aged 40-49 years.

Evidence suggests that TRT offers the greatest benefit in patients with biochemically confirmed hypogonadism, typically defined as total testosterone levels below 300 ng/dL on at least two early-morning measurements (although thresholds vary between 280 and 350 ng/dL, depending on the guideline), in the presence of compatible symptoms. Documented clinical improvements include enhanced sexual function, gains in lean mass, reduction in fat mass, and metabolic benefits such as decreased waist circumference and improved insulin sensitivity, particularly when combined with structured lifestyle interventions. Additional benefits reported in select cohorts include improvements in bone mineral density, mood, energy, vitality, and, to a modest degree, cognition.

Safety data, primarily from randomized controlled trials and large observational studies, suggest no significant increase in major adverse cardiovascular events when patients are carefully selected and appropriately monitored. Nonetheless, vigilance is required for erythrocytosis, prostate health, and fertility suppression. Long-acting intramuscular formulations carry the highest risk of hematocrit elevation, highlighting the importance of structured surveillance.

Current evidence supports a favorable risk-benefit profile for TRT in appropriately selected men in their 40s. However, important gaps remain regarding long-term, age-specific safety, optimal delivery methods, and precision medicine approaches. Future research should focus on age-stratified trials with extended follow-up to refine patient selection and optimize treatment strategies. In the meantime, individualized decision-making, guided by established clinical practice recommendations and regular monitoring, remains the cornerstone of safe and effective TRT in this demographic.

## Introduction and background

Background

Late-onset testosterone deficiency is increasingly recognized in early middle-aged men (40-49 years), driven by the interplay between physiological aging and modifiable comorbidities such as obesity, sleep apnea, and metabolic syndrome [1,2]. Estimates suggest that 6%-12% of men in this age group have serum testosterone levels below the reference range, and prescriptions for testosterone replacement therapy (TRT) have risen by more than 30% over the past decade [3]. Current guidelines emphasize that diagnosis requires both compatible clinical symptoms and consistently low morning testosterone levels. Consideration must also be given to assay variability, with recommendations favoring validated, high-precision techniques such as liquid chromatography-tandem mass spectrometry (LC-MS/MS) when available, alongside assessment of circulating sex hormone-binding globulin (SHBG) levels [2,3]. This is particularly important in men aged 40-49 years, where obesity, metabolic syndrome, and sleep apnea, which are highly prevalent in this demographic, can reduce SHBG concentrations and cause total testosterone to underestimate the true bioavailable fraction [3,4].

Rationale

Most studies on TRT combine middle-aged men with older adults, making it difficult to draw conclusions specifically for the 40-49 age group, which presents unique considerations regarding fertility, cardiovascular risk, and functional expectations. Evidence from clinical trials, meta-analyses, and recent reviews indicates that the most consistent benefits of TRT include improvements in sexual function, along with favorable effects on body composition (increased lean mass and reduced fat mass) and overall functional well-being [4,5]. However, effect sizes vary depending on baseline testosterone levels, treatment formulation, and adherence, and many studies disproportionately represent older populations [4]. More recent analyses contrasting pharmacological TRT with lifestyle and exercise interventions have further refined the interpretation of these benefits, highlighting the need to align therapeutic expectations with realistic goals for men in their 40s and to avoid inappropriate extrapolations from older cohorts [5].

This age group warrants particular attention because men in their 40s often retain fertility potential, making the suppressive effect of TRT on spermatogenesis more clinically relevant than in older adults. In addition, they typically present with fewer chronic comorbidities, which may influence both the safety profile and the magnitude of benefits observed with therapy. Moreover, men in their 40s frequently seek treatment with higher expectations of maintaining physical performance and long-term vitality, underscoring the importance of age-specific evaluation rather than reliance on data derived from older populations.

The TRAVERSE randomized controlled trial, which enrolled 5,246 men aged 45-80 years with a median follow-up of approximately 33 months, found no significant increase in major adverse cardiovascular events with TRT compared to placebo. However, numerical increases were observed in atrial fibrillation, acute kidney injury, and pulmonary embolism [1]. In 2025, the US Food and Drug Administration (FDA) updated the labeling of all testosterone products, removing prior language suggesting an increased cardiovascular risk and adding warnings regarding blood pressure effects, based on TRAVERSE and other evidence [3]. Erythrocytosis remains the most frequent dose-dependent adverse effect; current guidelines recommend regular monitoring of hematocrit, with dose adjustment or therapeutic phlebotomy when threshold values are exceeded [2].

Objective

To our knowledge, few reviews have specifically examined the indications, outcomes, and mid-term safety (12-36 months) of TRT in men aged 40-49 years. This narrative review aims to synthesize the current evidence on the indications, benefits, and risks of TRT in this demographic, offering an updated framework to guide personalized clinical decision-making.

## Review

Methods

Literature Search

A comprehensive literature search was conducted in PubMed/Medline, Embase, Scopus, and the Cochrane Library to identify publications on TRT in men aged 40-49 years. The search covered articles published between January 1, 2013, and August 1, 2025.

The strategy combined Medical Subject Headings (MeSH) and free-text terms with Boolean operators. For example, the PubMed search string was: ("testosterone replacement therapy"[MeSH Terms] OR "testosterone therapy" OR "androgen replacement") AND ("middle-aged men" OR "men in their 40s" OR "age 40-49" OR "early middle age") AND ("hypogonadism" OR "testosterone deficiency") AND ("safety" OR "adverse effects" OR "complications" OR "body composition" OR "sexual function").

No language restrictions were applied during the initial search; however, only studies published in English or Spanish were included. In addition, reference lists of eligible articles, relevant meta-analyses, and clinical guidelines from the Endocrine Society, American Urological Association (AUA), and European Association of Urology (EAU) were manually screened. All results were imported into EndNote X9 (Clarivate, London, UK) for reference management, and duplicates were removed prior to screening.

Selection Criteria

Inclusion criteria were as follows: (1) peer-reviewed articles published in English or Spanish; (2) studies including men aged 40-49 years, or those in which results for this subgroup could be extracted; (3) eligible study designs including randomized controlled trials, prospective or retrospective cohort studies, case-control studies, cross-sectional studies, narrative reviews, meta-analyses, and clinical practice guidelines; and (4) outcomes reporting at least one of the following: indications for TRT, clinical benefits (sexual function, energy, body composition, bone health, psychological well-being), or safety outcomes (hematological, cardiovascular, prostate, or fertility-related).

Exclusion criteria were as follows: (1) studies focused exclusively on men aged ≥50 years or ≤39 years without stratified results for the 40-49 age group; (2) studies evaluating testosterone use for bodybuilding or athletic performance without a medical indication; (3) animal or in vitro studies, conference abstracts without full-text availability, editorials, and opinion pieces lacking primary data or comprehensive synthesis; (4) studies investigating selective estrogen receptor modulators (e.g., clomiphene citrate) or gonadotropins (e.g., human chorionic gonadotropin), as these agents stimulate endogenous testosterone rather than provide replacement therapy.; and (5) articles in languages other than English or Spanish for which no translation was available.

Data Extraction and Synthesis

Two reviewers (Reviewers A and B) independently screened titles, abstracts, and full texts to determine study eligibility. Disagreements were resolved through discussion and consensus; a third reviewer was not required, as inter-reviewer agreement was high. Formal kappa statistics were not calculated. Data extraction was conducted independently by both reviewers using a standardized Excel form (Microsoft Corp., Redmond, WA, USA). Extracted information included the following: (1) study characteristics: author(s), year of publication, country, study design, and sample size; (2) population details: age range, diagnostic criteria for hypogonadism, baseline testosterone levels, and comorbidities; (3) intervention characteristics: TRT formulation (intramuscular, transdermal, oral), dosage, treatment duration, and monitoring protocol; and (4) outcomes assessed: (a) indications and clinical/biochemical criteria for TRT initiation; (b) clinical benefits, including changes in sexual function, energy, body composition, bone mineral density, and psychological well-being); and (c) safety outcomes, including hematological changes (hematocrit, hemoglobin), cardiovascular events, prostate-specific antigen (PSA) levels, fertility parameters, and other adverse effects.

Although formal risk-of-bias tools (e.g., GRADE, Cochrane RoB) were not applied, study quality was considered by prioritizing randomized controlled trials, meta-analyses, and clinical guidelines, with observational studies used to provide real-world context. In cases of conflicting evidence, higher-level data (randomized controlled trials and meta-analyses) were given precedence, and discrepancies were explicitly reported and discussed to ensure a balanced interpretation. The synthesis was thematic, structured into three main sections: diagnosis and indications, clinical outcomes, and mid-term safety.

Central body

Evidence Synthesis From Included Studies

Nine studies met the eligibility criteria for inclusion (Table 1), providing a heterogeneous but complementary perspective on TRT in men aged 40-49 years. The relatively small number reflects the deliberate focus on biochemically confirmed hypogonadism and age-specific outcomes, distinguishing this review from broader analyses that combine middle-aged and older populations. Although most trials included wider age ranges, subgroup analyses and stratified reporting allowed us to extract data relevant to men in their 40s, ensuring that conclusions reflect outcomes specific to this group. Improvements in sexual function were the most consistently reported outcome across studies. However, these findings are based on descriptive synthesis rather than pooled statistical estimates, as heterogeneity in study design and outcome reporting precluded formal meta-analysis with confidence intervals [6-9]. Several studies also reported favorable changes in body composition, including increased lean body mass (LBM) and reduced fat mass, particularly with intramuscular and transdermal formulations [8,10]. Effects on psychological well-being and energy were more variable, with improvements generally limited to patient-reported outcomes such as reduced fatigue and enhanced mood, but lacking consistent objective measures to define clinically meaningful change.

**Table 1:** Comparative summary of trials and reviews on testosterone replacement therapy in middle-aged men T: testosterone, T2DM: type 2 diabetes mellitus, CVD: cardiovascular disease, TRT: testosterone replacement therapy, IM: intramuscular, RCT: randomized controlled trial, Hct: hematocrit, PSA: prostate-specific antigen, MACE: major adverse cardiovascular events, AF: atrial fibrillation, AKI: acute kidney injury, PE: pulmonary embolism, LBM: lean body mass.

Study/author	Age group 40-49 available?	Study design	Sample size	Diagnostic criteria	Baseline T levels	Comorbidities	TRT formulation	Duration	Monitoring	Main benefits	Main safety findings
Lincoff et al. (2023) [1] - TRAVERSE trial (NEJM)	Yes (45-49 subgroup)	Multicenter RCT	5246	Total T < 300 ng/dL + symptoms	227 ± 48 ng/dL	T2DM, CVD risk, obesity	Transdermal gel 1.62%	~33 months	Hct, PSA, lipids, serum T	↑ sexual function, ↓ anemia, ↑ lean mass	No ↑ MACE; ↑ AF, AKI, PE; erythrocytosis (Hct > 54%)
Bhasin et al. (2024) [6] - TRAVERSE substudy (Prediabetes/DM)	Yes (45-49 subgroup)	Multicenter RCT	5204	Total T < 300 ng/dL + symptoms	227 ± 48 ng/dL	T2DM, CVD risk, obesity	Transdermal gel 1.62%	~33 months	Hct, PSA, lipids, serum T	No significant change in diabetes progression/remission	Same as TRAVERSE
Bhasin et al. (2024) [7] - TRAVERSE substudy (Depression)	Yes (45-49 subgroup)	Multicenter RCT	5204	Total T < 300 ng/dL + symptoms + depression criteria	227 ± 48 ng/dL	T2DM, CVD risk, obesity, depression	Transdermal gel 1.62%	~33 months	Hct, PSA, lipids, serum T	↑ energy, ↑ sleep, ↑ cognition in depressive subgroup	Same as TRAVERSE
Hudson et al. (2023) [8] - Lancet Healthy Longev	Yes (≥40, subgroups)	Secondary analysis	~2000	Low T + symptoms	Not reported	Obesity, metabolic syndrome	Variable (not specified)	Variable	Variable	↑ sexual function regardless of age/T level	Not specified
Gianatti et al. (2014) [9] - Eur J Endocrinol	Yes (middle-aged men)	Narrative review	—	Guideline-based	—	—	Variable	—	—	Summary of benefits and limitations	Hematologic, CV, prostate risks
Borst and Mulligan (2007) [10] - Clin Interv Aging	Yes (≥40 included)	Meta-analysis of RCTs	11 RCTs	Trial-specific criteria	Variable	Variable	IM and transdermal	Variable	Variable	↑ lean mass, ↑ muscle strength	Moderate erythrocytosis
Saad et al. (2016) [11] - obesity cohort	Yes (≤65, includes 40-49)	Prospective cohort	156	Total T < 12.1 nmol/L + symptoms	Not reported	Obesity, metabolic syndrome	IM testosterone undecanoate	Up to eight years	Hct, PSA, serum T	↓ weight, ↓ waist circumference, ↑ metabolic profile	No ↑ major CV events
Snyder et al. (2016) [12] - NEJM	Yes (middle-aged men)	Narrative review	—	Guideline-based	—	—	Variable	—	—	Sexual, bone, anemia benefits	Hematologic, CV, prostate risks
Mulhall et al. (2018) [13] - AUA Guideline (J Urol)	Yes (45-49 subgroup)	Expert commentary	—	Same as TRAVERSE	—	—	Same as TRAVERSE	—	Same as TRAVERSE	Reinforces TRAVERSE benefits and safety	Same as TRAVERSE

From a safety perspective, the TRAVERSE trial [6] and its secondary analyses [11,12] reported no statistically significant increase in major adverse cardiovascular events (MACE) in men receiving TRT compared to placebo over a median follow-up of 33 months, although numerical increases in atrial fibrillation, acute kidney injury, and pulmonary embolism were observed. Hematological monitoring remains critical, as erythrocytosis is the most frequent dose-dependent adverse effect, with hematocrit exceeding recommended thresholds in up to 18% of treated participants in some trials [13]. Notably, the occurrence of adverse events has been linked to the testosterone levels achieved, with both overtreatment (supraphysiological levels) and undertreatment (subtherapeutic levels) contributing to the variability in reported safety outcomes.

Although few studies stratified results specifically for men in their 40s, indirect evidence suggests that this age group may experience proportionally greater improvements in body composition, metabolic profile, and sexual health compared with older cohorts, reflecting fewer comorbidities and higher baseline functional capacity [8,10]. When therapy is appropriately indicated, closely monitored, and aligned with patient goals, the overall risk-benefit balance appears favorable in this demographic. Taken together, the available evidence supports TRT as a viable option for well-selected men aged 40-49 years with clinically significant symptoms and biochemically confirmed hypogonadism, provided that treatment is carefully individualized and follow-up is maintained to optimize both efficacy and safety.

Clinical Indications for TRT in Men Aged 40-49 Years

Analysis of the included studies indicates that TRT is most beneficial in men aged 40-49 years with biochemically confirmed hypogonadism, typically defined as total testosterone levels below 300 ng/dL on at least two early-morning measurements. Some guidelines recognize a diagnostic range between 280 and 350 ng/dL, provided compatible symptoms are present, such as reduced libido, mild erectile dysfunction, or persistent fatigue. Because these symptoms may also overlap with depression, sleep disorders, or age-related changes, careful differential diagnosis is essential [2,14]. Guideline bodies, including the Endocrine Society and the American Urological Association (AUA), consistently recommend that both biochemical and clinical evidence be established before initiating therapy [2]. In men with borderline total testosterone, assessing SHBG-adjusted free testosterone can improve diagnostic accuracy and prevent inappropriate exclusion of symptomatic patients [15,16].

When applied specifically to men in their 40s, these diagnostic criteria are associated with a particularly favorable risk-benefit profile. Several trials in our dataset reported clinically meaningful improvements in sexual function (IIEF score gains of 4-7 points), increases in lean mass (2-4 kg), and reductions in fat mass (2-3 kg) over 6-12 months of therapy, particularly with intramuscular formulations [14,15,17]. Beyond sexual health, metabolic benefits were also observed: the T4DM trial showed that combining TRT with structured lifestyle interventions reduced progression to type 2 diabetes by 40% while simultaneously improving body composition metrics [16].

Bone health benefits, often underrecognized in younger men, are also noteworthy. A mid-term trial of testosterone undecanoate in men with late-onset hypogonadism and metabolic syndrome reported annual bone mineral density gains of about 5%, an effect size comparable to first-line osteoporosis therapies [17]. Pooled analyses further suggest modest but consistent improvements in mood, vitality, and cognitive performance, particularly in men with coexisting metabolic syndrome or depressive symptoms [18].

Taken together, the evidence supports a broader, yet still selective, application of TRT in men aged 40-49 years. While the primary indication remains symptomatic biochemical hypogonadism, additional considerations, such as metabolic risk reduction, early bone preservation, and mood enhancement, may provide clinically defensible reasons to initiate therapy in this group. This nuanced approach, anchored in guideline-based practice and reinforced by emerging trial data, highlights the importance of individualized assessment rather than rigid exclusion by age.

Safety Considerations of TRT in Men Aged 40-49 Years

Safety remains a key determinant in initiating TRT, particularly in relatively younger men for whom long-term exposure is anticipated. Data from the TRAVERSE trial and subsequent secondary analyses confirm that TRT does not significantly increase the incidence of MACE compared with placebo in men aged 45-80 years, with follow-up durations in most trials ranging from 12 to 36 months, informing mid-term but not long-term safety [1,14,19]. Although the mean age in TRAVERSE exceeded 60 years, limiting direct extrapolation to men aged 40-49, subgroup analyses and smaller RCTs in younger cohorts suggest a similarly neutral cardiovascular risk profile when patients are carefully selected and comorbidities appropriately managed [20,21]. However, slight numerical increases in atrial fibrillation, pulmonary embolism, and acute kidney injury have been reported, underscoring the importance of vigilant monitoring, including regular assessment of serum testosterone to confirm therapeutic range, hematocrit and hemoglobin to detect erythrocytosis, and PSA levels to ensure prostate safety [14,20].

Erythrocytosis remains the most frequent dose-dependent adverse event, with prevalence ranging from 6% to 18% depending on the formulation, particularly with long-acting intramuscular testosterone undecanoate. Current guidelines recommend monitoring hematocrit at baseline, again at 3-6 months after initiation, and annually thereafter, with therapy adjustments or phlebotomy if hematocrit exceeds 54% (≥52% in some guidelines) [2,22,23].

In addition to routine surveillance, clear protocols for managing adverse events are essential for the safe use of TRT in men aged 40-49 years. For erythrocytosis, guidelines recommend withholding therapy or reducing the dose if hematocrit exceeds 54%, with therapeutic phlebotomy as an appropriate option in persistent cases [2,22]. Prostate safety requires baseline and follow-up PSA testing, with suspension of TRT and referral for urological evaluation if PSA exceeds 3 ng/mL or if digital rectal examination findings are abnormal [23,24]. In men seeking to preserve fertility, exogenous testosterone should be avoided; alternatives such as clomiphene citrate or hCG may be considered to maintain spermatogenesis [25,26]. These practical measures ensure that monitoring is both feasible and actionable, balancing therapeutic benefits with proactive risk mitigation.

Prostate health monitoring remains a cornerstone of TRT safety. Current evidence does not show an increased incidence of prostate cancer in hypogonadal men receiving therapy; however, baseline PSA testing and digital rectal examination are recommended, followed by age-appropriate surveillance [23,24]. In men at higher baseline risk, for example, those with PSA > 3 ng/mL in combination with additional risk factors (acknowledging that thresholds may vary by age-adjusted norms), TRT should be deferred pending urological evaluation [2,24].

In the context of fertility, exogenous testosterone suppresses spermatogenesis by downregulating gonadotropin secretion, an effect particularly relevant to men in their 40s who may still be planning paternity, as more than 90% develop oligospermia or azoospermia during treatment [25,26]. Alternatives such as clomiphene citrate or hCG should be considered for men wishing to preserve fertility. Clomiphene citrate may be especially attractive in men with reasonably normal LH, as it maintains spermatogenesis, is relatively simple to dose, and avoids suppression of the hypothalamic-pituitary-gonadal axis inherent to exogenous testosterone. Although concerns about long-term outcomes remain, its clinical use is increasing, and it deserves consideration as a viable first-line option in this subset [26].

Overall, the safety profile of TRT in appropriately selected men aged 40-49 years appears favorable, provided treatment is coupled with systematic monitoring of hematological, cardiovascular, and prostate-related parameters.

Limitations and future directions

This narrative review has several limitations. First, as a narrative rather than a systematic review, the selection process may be subject to publication bias and the selective inclusion of studies aligned with prevailing hypotheses. Although a broad database search was performed, heterogeneity in study design, participant characteristics, TRT formulations, and outcome measures limits direct comparability and precludes formal meta-analysis [27,28].

Second, although this review focuses on men aged 40-49 years, a clinically underrepresented group, the evidence specific to this age range remains limited. Many RCTs and meta-analyses combine middle-aged and older populations, which may overestimate or underestimate both benefits and risks in younger men [29]. Furthermore, long-term safety data beyond 5-10 years of continuous TRT are lacking, particularly with respect to cardiovascular and prostate outcomes.

Third, there is a paucity of research on individualized treatment strategies, including the influence of genetic markers, baseline metabolic profiles, and androgen receptor polymorphisms on therapeutic response. Likewise, the comparative effectiveness of different delivery methods (intramuscular, transdermal, oral) in this specific age group remains insufficiently explored [11].

In addition, translating trial evidence into routine practice presents challenges. Guideline-recommended monitoring may be difficult to sustain due to patient adherence, physician workload, and healthcare system constraints. Adverse events are often attributed directly to testosterone, leading to discontinuation, while patients perceiving clear benefits tend to request continuation, and those with limited benefit frequently discontinue on their own. These real-world dynamics highlight the gap between controlled trial conditions and daily clinical care.

Future research should prioritize large, age-stratified RCTs with extended follow-up to clarify long-term safety and refine patient selection. Incorporating precision medicine approaches and real-world registry data could help bridge current gaps and support more tailored, evidence-based TRT protocols for men in their 40s.

## Conclusions

Building on the findings discussed, the present review suggests that TRT in carefully selected men aged 40-49 years with documented biochemical hypogonadism and compatible symptoms may provide meaningful benefits across multiple health domains. Reported improvements in sexual function, lean mass, fat distribution, bone density, metabolic risk, and mood highlight its therapeutic potential, though variability across studies warrants cautious interpretation. These possible advantages depend on strict patient selection, close monitoring, and adherence to established clinical guidelines. Safety considerations, particularly erythrocytosis, prostate health, and cardiovascular events, remain central. The available evidence indicates that, when guidelines are followed and individualized risk assessments are applied, the balance in this age group tends to favor benefit over risk.

Nonetheless, current literature still lacks robust long-term, age-specific data on safety and efficacy. Addressing these gaps through well-designed, stratified clinical trials will be essential to refining treatment protocols. Until such evidence emerges, clinicians should adopt a personalized approach, integrating patient goals, comorbidities, and continuous reassessment to optimize outcomes while minimizing risks. Importantly, while mid-term safety data are reassuring overall, most outcomes beyond hematocrit monitoring lack causal certainty, as hypogonadal populations may differ from controls in both measured and unmeasured ways. This underscores the need for larger, longer, and age-stratified trials to delineate safety signals and prevent misinterpretation of interim findings.
